# Abstracts

**Published:** 1893-06

**Authors:** 


### Abstracts.

### THE NERVOUS AFFECTIONS THAT MAY ARISE FROM MALARIA.

By WILLIAM BROWNING, M. D., Brooklyn, N. Y.

Of the various toxic agents, including thereunder infections as well as purely chemical poisons, that often attack the nervous system, malaria is one of the more common, the relative frequency, however, varying greatly with time and place. These parallel causes produce more or less comparable series of cases.

By malaria I mean not simply mal-aria, or bad air, but a distinct and infectious disorder.

It is especially in the larvated and chronic forms of intermittent poisoning that the nervous system becomes prominently involved. In such cases the patient may seek advice for the nervous affection simply. The true cause can then only be arrived at indirectly, and often one feels the lack of safe clinical guides. The blood-changes that in typical ague may be utilized for certainty in diagnosis, are, so far as we know, not present, or at least not easily demonstrated here.[1] Where exacerbations or remissions occur, the diagnosis is much assisted; but for the most part these are continuous conditions, and many of the manifestations are of secondary origin; hence the personal equation of the observer may affect somewhat the trustworthiness of the etiological diagnosis. Many of the severest cases seen in Brooklyn have contracted their infection elsewhere; still those of home origin are ample in number and variety.

Though no recent or comprehensive review of this subject seems to have been made, it is customary and convenient to classify such cases according to the part of the nervous system specially attacked.

*A.* Brain.

*B.* Spinal Cord.

*C.* Peripheral Nerves.

*A.* The brain troubles attributed to this cause include: Intracranial inflammations of a meningitic type, mental disorders, pig-

1. In Da Costa's severe case, ("Malarial Paralysis." *Internat. Clinics*, Oct., 1891,) characteristic forms were found.

mentary deposits,[1] epilepsy, chorea, aphasic symptoms, hysteria (Regnault's case, Gaz. des Hôp., 1890, No. 3), neurasthenia and hystero-neurasthenia (Teissier's two cases, *Bull. Med.*, Paris, 1890, iv., 397-9).

. . . . . . . . . . . . . . . . . . . . .

B.—SPINAL TROUBLES.

It is not as yet established that these are ever of malarial origin, although Morton Prince, of Boston, (*Jour. Nerv. and Ment. Dis.*, 1889, Oct.,) believes it for certain cases of tabes and disseminated sclerosis. Torti and Angelini have also recently described chronic malarial infection with symptoms of sclerosis en plaques. In this respect the paludal poison is comparable to alcohol. It affects preferably the peripheral nerves, but in many cases the brain is either also involved to a limited extent or may alone suffer. For some unexplained reason the cord seems to have a remarkable immunity of both these agents. In various spinal cases one finds a past history of malaria, but not closely enough connected with the myelitis to be considered the cause.

It has been suggested that cerebro-spinal meningitis might be of miasmatic origin. A boy of five years, living beside Gowanus Canal, seen with Dr. Maddren in April of this year, was sick several weeks with variable fever, general tenderness, distinct opisthotonos, etc. He did best on anti-malarials, and finally recovered, but it was not a clear case. Dr. Bartley gives the following notes of a more definite case in a boy of three years: He was seen by several competent men and considered to be suffering from sporadic cerebro-spinal meningitis. There was wry-neck, pain on motion of head, tenderness along post-cervical region and on percussing over the spleen, low fever, tache cerebrale, anemia, etc. Under quinine he recovered completely in a week. In such forms as this there can only be a pseudo-meningitis, but its occurrence may explain some of the so-called sporadic cases.

. . . . . . . . . . . . . . . . . . . . .

C.—PERIPHERAL NERVES.

Of this class are a majority of the nerve cases from malaria as seen in general practice. Most of these fall under one of two

1. The question of pigment emboli and deposits is essentially one of pathology, though Hammond (Trans. Am. Neurolog. Assoc., 1875,) and others have sought to deduce their clinical bearing. Councilman and Abbott (Am. Jour. Med. Sci., 1885, April,) have described these in connection with hyaline bodies, but interest of late has shifted to the subject of microörganisms.

clinical types: (*a*) Neuralgia, (*b*) Neuritis, although (*c*) Contractures, and (*d*) Paralysis appear also to occur as independent manifestations.

That neuralgia is frequently due to this cause is so well known that, though a very important fact, it need be but briefly considered. In neuralgia remissions are the rule, whilst in neuritis any such feature is necessarily obscured. Doubtless the neuralgia is often but the first stage of neuritis. Whilst, perhaps, any nerve in the body may be attacked, there are certain favorites. Amongst neuralgias brow-ague and sciatica are best known, though in Brooklyn, at least, other forms are common. One case of severe cruralgia (left anterior) was in a night engineer employed in the basement of a New York printing establishment (seen in January, 1888; has remained free since). Visceral neuralgias, taking the form of colic, are often very severe, as in a recent case contracted in West Florida. Another form in a colleague (living on the line of the great storm-sewer excavation) was located by him deeply beneath the bridge of the nose, and a lighter similar case might be added. Various headaches, more or less migrainous in character, are common, and, unfortunately, these may continue later as a less frequent hemicrania or migraine. In one case, (wife of the just-mentioned colleague,) where the brachial plexus with the arm-nerves was the seat, tender points became established at neck, shoulder and elbow. In the preradial region the pain became especially severe. Reddish papules, with incipient ulceration, appeared over the ball of the thumb, and a couple of the former even up the arm. This cutaneous trouble was possibly herpetic in its nature. The whole yielded promptly to systemic remedies.

. . . . . . . . . . . . . . . . . . . . . .

In conclusion, it may be well again to remark that malaria is but one of the various causes of nervous diseases. There is a justifiable aversion to the over-frequent assumption of a malarial influence; still this need not blind us to present facts. A more careful scrutiny of our cases may show that at certain times, and in certain places, these nervous manifestations from malaria are far from uncommon.—*Brooklyn Medical Journal*, January, 1893.

---

Goodell says: A nervous bladder is one of the earliest symptoms of a nervous brain; for nervousness means a deficient control of the higher nerve centers over the lower ones; the vesical irritability indicates a lack of brain control.—*Practitioners' Monthly*.

### ARE ASYLUM PHYSICIANS PARTY PENSIONERS?

(From the *American Journal of Insanity*, April, 1893.)

The notion that public officers are the pensioners of a party, not the servants of the whole people, seems to die hard. The prospect appears to be that the officers of all the hospitals for the insane of the State of Illinois will be turned out to make room for members of the political party which, after an outing of thirty-five years, has once more gained the upper hand. It is true that the present Governor, in his canvass, made charges of extravagance and mismanagement against those institutions, but we presume that no one will seriously maintain that a lack of confidence in their management is the only, or even the principal, reason for so sweeping a change. It is also true that, so far as one wrong can justify another, the course of the Republican party, during the long period of its dominance in the State, has afforded an excuse for such a course. Only Republicans have been appointed on the boards of trustees, and we understand that the officers of the hospitals have been regularly assessed a portion of their salaries for the campaign funds. It is not long since the superintendent of the hospital at Anna was driven out of office with little or no pretense of concealment of the fact that the ground of his dismissal was his lukewarmness in partisanship, and, from all that we can learn, his successor has not erred in that direction, although his attainments as an alienist have not, we believe, even yet earned him any very wide celebrity.

We do not suppose that if a member of Governor Altgeld's family were to become insane, and he were looking for a suitable private hospital, it would ever occur to him to enquire into the physicians' views on the tariff. We have no doubt that multitudes of those who will applaud his action in this matter, or take it as a matter of course, employ, by choice, physicians of a different political faith from their own in their families, and would laugh at the idea that a man's political views have anything to do with his professional competency. It is the view that the salaries of these officers are not, primarily, the reasonable compensation for honest and faithful discharge of their duties, but the reward of activity in an entirely different field, that allows people to view with approval or indifference such changes, entirely without regard to the merits either of those who are turned out or those who are put in.

The pernicious effect of such a policy is so plain that we should feel as if we were insulting the intelligence of our readers by arguing the question. Men whose aspirations are for professional eminence and usefulness will hesitate about accepting positions in which such qualities count for nothing. Even if competent men are secured, they are sure, in a State in which parties are pretty evenly balanced, under such a system, to be turned out before they have acquired the experience that will enable them to do their best work. The inevitable tendency, under such conditions, is to the filling of the offices by men whose only object is to make money out of them, and who, knowing that the time is short, will "make hay while the sun shines."

We have no doubt that, in time, the mischief of treating the funds provided for the relief of the unfortunate as plunder will become so plain that it will be no longer possible in a government like ours. But we fear that a good many object lessons will be needed first, and in the meantime the insane must suffer. We shall be as much surprised as gratified if the medical profession of Illinois, without distinction of party, shall denounce the iniquity as it deserves. In the meantime, we believe it is the right and the duty of the American Medico-Psychological Association to scan critically the qualifications of the men who profit by the misfortunes of its honored members, should they apply for admission.

---

### THE MANAGEMENT OF SUPPURATION COMPLICATING TUBERCULOUS DISEASE OF THE BONES AND JOINTS.

By V. P. GIBNEY, M. D., New York.

In concluding this desultory paper, let me summarize as follows:

1. Protect the joint, about which the bone lesion exists, in the early stage and in the later stages, whether the abscess is let alone, aspirated, or incised.

2. In cases where the suppurative process is confined to a small area, it is good surgery to leave the small abscesses alone if the protective appliance is adequate.

3. It is good practice to aspirate where the abscess is in the way of the proper adjustment of apparatus, and by such procedure one may expect good results in at least fifty per cent.

4. The simple incision of an abscess dependent upon bone disease depends for good result upon the extent of the bone lesion.

5. Excision of the hip is not a measure to be employed in all cases where extensive suppuration exists, but must depend largely upon the condition of the patient and the location and extent of the abscesses.

6. Expectant treatment for the knee and ankle joint in children yields the best results for life and limb.

7. Amputation of the ankle in a child is rarely ever justifiable; of the knee is justifiable only when amyloid disease of liver or kidneys threatens, or is present; of a hip, after a thorough excision has failed.

8. The long continued employment of a good fitting splint to the back in Potts' disease of the spine, will yield better results than any operative procedures on the bone with which I am familiar.—*Southern Medical Record.*

---

### THE ADVANTAGES OF A PHYSICIAN DISPENSING HIS OWN MEDICINE.

BY CARYL B. STORRS, M. D.

THIS is a subject which has been discussed to a great extent, both pro and con, for a long time. The principal reasons which have been given in favor of every physician being his own druggist, may be mentioned as follows:

1. To make a physician more independent.
2. On account of the habit of substitution prevalent among certain druggists.
3. To make sure of exhibiting pure and fresh drugs.
4. To prevent refilling of prescriptions without the physician's knowledge.
5. To show his disapproval of the practice of counter prescribing and the selling of patent medicines.
6. To center the pecuniary profits upon the physician.
7. To assure cleanliness in the preparation of medicines.
8. To educate the doctors to practical pharmacy.
9. To inspire confidence in the patient.
10. To check the growing use of proprietary medicine by the profession.—*The Medical Age*, March, 1893.

---

### DUBOISINE IN PARALYSIS AGITANS.

PROF. MENDEL, of Berlin, (*La Semaine Médicale*, No. 10, 1893,) has obtained a notable diminution of the tremor, in cases of paralysis agitans, from the hypodermic injection of duboisine, in doses of two to three decimilligrammes ($\frac{1}{320}$ to $^{20}{}_{0}$ gr.) He tried it in twelve cases ; in none of them did the drug fail to act. A quarter of an hour after injection the trembling of the hands diminished, so that the patient could write legibly; walking also became easier. The sedative action lasts from three to five hours, with the same dose repeated. In this manner a paralytic with three injections is able to pass a day without violent trembling. An evening injection will enable a patient with insomnia to sleep the night through. Its action upon the patient's speech varies ; sometimes it is favorable, then again the contrary. He regards duboisine as the most efficacious remedy for the tremor of this disease. It has the advantage over hyoscine of being less toxic and more efficacious.—*Cincinnati Lancet-Clinic.*

---

### SOME REASONS FOR DAILY EXERCISE.

1. Any man who does not take time for exercise will probably have to make time to be ill.

2. Body and mind are both gifts, and for the proper use of them our Maker will hold us responsible.

3. Exercise gradually increases the physical powers and gives more strength to resist sickness.

4. Exercise will do for your body what intellectual training will do for your mind—educate and strengthen it.

5. Plato called a man lame because he exercised the mind while the body was allowed to suffer.

6. A sound body lies at the foundation of all that goes to make life a success. Exercise will help to give it.

7. Exercise will help a young man to lead a chaste life.

8. Varied, light and brisk exercise, next to sleep, will rest the tired brain better than anything else.

9. Metal will rust if not used, and the body will become diseased if not exercised.

10. A man "too busy" to take care of his health is like a workman too busy to sharpen his tools.—*Glasgow Herald.*

---

Curious Facts.—The year of greatest growth in boys is the seventeenth, in girls the fourteenth. While girls reach their height in their fifteenth year, they acquire full weight at the age of twenty. Boys are stronger than girls from birth to the eleventh year ; then girls become superior physically to the seventeenth year, when the tables are again turned and remain so.—*Ex.*

